# Prevalence of Coccidiosis in Broiler Chicken Farms in Western Iran

**DOI:** 10.1155/2014/980604

**Published:** 2014-09-10

**Authors:** Jamal Gharekhani, Zivar Sadeghi-Dehkordi, Mohammadali Bahrami

**Affiliations:** ^1^Department of Parasitology, Central Veterinary Laboratory, Iranian Veterinary Organization, Hamedan Veterinary Office, Mellat Street, Hamedan, 6519611156, Iran; ^2^Department of Parasitology, Faculty of Paraveterinary Medicine, Bu-Ali Sina University, Hamedan, Iran; ^3^Department of Poultry Disease, Iranian Veterinary Organization, Hamedan, Iran

## Abstract

The main goal of current study was to investigate the prevalence of coccidiosis in broiler farms in Hamedan province, western Iran. Chicks and fecal samples were collected in all of the 220 broiler farms in this region. All viscera were examined for gross pathological changes. The mucosa of small intestine and the caeca were examined for the presence and identification of parasitic forms using parasitology methods. The overall rate of coccidiosis was 31.8%; *E. acervulina* (75.7%), *E. tenella* (54.3%), *E. necatrix* (28.6%), and *E. maxima* (20%) were determined. Mixed infections were observed in all of the positive farms. There was a statistical significant difference (*P* < 0.05) among infection rate and age groups, dysentery, history of colibacilosis and clostridiosis in farm, and history of coccidiostats consumption, unlike to breed (*P* > 0.05). This is the first report of coccidiosis rate in broiler farms in this region. Further additional researches and design control strategies for improving management in farms are necessary.

## 1. Introduction

Coccidiosis is caused by various species of* Eimeria*, an Apicomplexa protozoan parasite. It is one of the common diseases in poultry, which is responsible for major economic losses worldwide [1, 2].

The disease occurs only after ingestion of sporulated oocysts in susceptible hosts. Both clinically infected and recovered birds shed oocysts in their droppings, which contaminate feed, dust, water, litter, and soil. Oocysts may be transmitted by mechanical carriers (e.g., equipment, clothing, insects, farm workers, and other animals) [3].

Coccidiosis occurs in the epithelial cells of the intestine, despite the advances in nutrition, chemotherapy, management, and genetics [3].* E. tenella* and* E. necatrix* are the most pathogenic and cause bloody lesions, high morbidity, and mortality [4, 5]. Most* Eimeria* spp. affect birds between 3 and 18 weeks of age and can cause high mortality in young chicks. Mixed infections are commonly found under field conditions [5, 6].

Coccidiosis in poultry is characterized by dysentery, enteritis, emaciation, drooping wings, and poor growth. Feed and water consumption are depressed. Weight loss, development of culls, decreased egg production, and increased morbidity and mortality may accompany outbreaks [7]. Bad management, such as wet litter that encourages oocyst sporulation, contaminated drinkers and feeders, bad ventilation, and high stocking density can exacerbate the clinical signs [6].

Coccidiosis is still a major problem worldwide; it is due to the difficult diagnosis. Identification of different species based on morphology of oocysts is very challenging and requires expertise. A diagnosis of clinical coccidiosis is warranted if oocysts, merozoites, or schizonts are seen microscopically and if lesions are severe [8].

Knowledge regarding the farm conditions is necessary for developing the best prevention program, enabling the recognition of factors that influence the possibility of incidence of the disease [5, 9]. The role of coccidiosis in economic losses of poultry is not clearly recognized in Iran. The epidemiological studies on poultry coccidiosis are known in only limited scale in Iran [1–3, 9]. However, there is no published data on coccidiosis in broiler chicks in western Iran.

The main aim of current study was to investigate the prevalence of coccidiosis in broiler farms in Hamedan province, western Iran.

## 2. Materials and Methods

### 2.1. Study Area

Hamedan province with mountainous and mild climate is located in western Iran (34.77°N and 48.58°E). It covers an area of 19,546 km² and average annual temperature is 11.3°C. This province is economically important for crops and animal husbandry such as poultry industry. Broiler chicks population in this area is approximately 2,000,000 in 220 farms. The method of housing the broiler is an intensive deep-litter system. The average age of slaughter and live weight are about 42 days and 2.2 kg, respectively.

### 2.2. Sampling

This study was conducted in all of the 220 broiler farms in Hamedan province. In cross-sectional study during March to September 2013, each farm was visited once during the rearing period and five chicks from each house located in these farms were taken randomly. Also, five fecal samples were collected around the drinker and feeders of the same house from which chickens were collected on each farm [2]. All of the farms had no history of vaccination against coccidiosis. The chicks and fecal samples were brought to the laboratory for necropsy and parasitology examination, respectively.

Information regarding the age (<4, 4–6, and >6 weeks) and breed of chicks, history of dysentery, colibacilosis, clostridiosis, and coccidiostats consumption in flock and general information of farms such as farmer's name, address, and farm location were collected through questionnaires [Table 1].

### 2.3. Sample Examination

The chicks were subjected to routine postmortem examination. All viscera were examined for gross pathological changes. Mucosal scrapings of small intestine and the caeca were made and examined microscopically for the presence and identification of oocyst and asexual forms of* Eimeria*.

The presence of oocyst in the fecal samples was examined by the flotation method using saturated solution of sugar [2, 7]. The modified McMaster technique was used to quantify the oocyst. For sporulation, positive samples were placed in Petri dishes, conditioned with a solution of 2.5% potassium dichromate at room temperature, and aired daily for up to two weeks [10]. The* Eimeria* spp. were determined based on morphology of oocysts and sporocysts (shape, color, form index, micropyle and its cap, and presence or absence of residual) and time of sporulation. There were at least 10 morphologically characterized oocysts of each species for identification [8, 10].

### 2.4. Statistical Analysis

Statistical analysis was performed by using the software package SPSS version 16.0 for Windows. The differences among variables were evaluated by chi-square test. A *P* value ≤ 0.05 was considered statistically significant.

## 3. Results and Discussion

The prevalence rate of coccidiosis was determined to be 31.8% (Table 1); this rate is low compared to investigations in Iran and other countries. In positive samples, the number of oocysts was found to be between 110 and 1,800.

In previous studies, the infection rate was reported to be 54.3% in Turkey [11], 20.6% and 70.9% in Ethiopia [4, 12], 31.7% and 39.6% in India [7, 13], 36.7% and 52.9% in Nigeria [14], 71.9% in Pakistan [15], 78% in Jordan [6], 88.4% in Argentina [16], and 92% in Romania [5]. In Iran, this rate was reported to be 75% in North, 64% in Southwest, 55.96% in Northwest, and 38% in Northeast regions [1–3, 9].

The highest rate of* Eimeria* spp. was determined* E. acervulina* (75.7%), followed by* E. tenella* (54.3%),* E. necatrix* (28.6%), and* E. maxima* (20%) (Table 2). Mixed infections with two or more* Eimeria* spp. were observed in all of the positive farms. In the studies from North, Northeast, and Northwest of Iran, five, three, and five* Eimeria* spp. were identified, respectively [1, 2, 9]. In our work,* E. acervulina* was the most prevalent species, which is in agreement with several previous studies in Europe, Australia, North America, and Iran [1, 2, 5, 9]. In Hadipour et al. [3] study in Southwest of Iran, four* Eimeria* spp. were identified;* E. tenella* was the most prevalent. Our findings are in parallel with reports from Sweden, France, Argentina, and Jordan suggesting that detected species of* Eimeria* are widespread in most countries [6, 16–18].

In current investigation, there was a statistical difference among coccidiosis rate and age groups (*χ* ^2^ = 9.719, df = 2, *P* = 0.007), dysentery (*χ* ^2^ = 42.021, df = 1, *P* < 0.0001), history of colibacilosis in flock (*χ* ^2^ = 67.117, df = 1, *P* < 0.0001, odds  ratio = 16.5), history of clostridiosis (*χ* ^2^ = 7.449, df = 1, *P* = 0.006, OR = 2.6), and history of coccidiostats consumption (*χ* ^2^ = 85.26, df = 1, *P* < 0.0001). The coccidiosis rate was reported to be 50% in Arbor-Acers-Plus breed, 33.3% in Cobb, and 29.4% in Ross, whereas the difference was statistically nonsignificant (*χ* ^2^ = 0.485, df = 2, *P* = 0.784) (Table 1).

Age is one of the most principal factors in coccidiosis [9]. In our study, the higher rate of coccidiosis was determined in >6 weeks' age groups; the significant relationship was observed in agreement with other researchers [1, 9, 12, 14, 15]. Muazu et al. [14] suggested that all ages of poultry are susceptible to infection but usually resolves itself around 6–8 weeks of age. It seems that the relationship between age and prevalence rate of coccidiosis is direct due to complete life cycle and the increase of oocysts consumption.

There was a significant difference in infection rateand use of coccidiostats. Our finding is incongruent with Nematollahi et al. [2] study and indicates the failure to control the disease using chemoprophylaxis under the rearing practices. This might be due to misuse of coccidiostats (dose or improper mixing in feed) or the development of resistance of local strain of* Eimeria* to variable compounds [3]. Over time, the coccidiostats have become less effective due to development of drug resistance. Drug resistance to* Eimeria* strains is responsible for subclinical coccidiosis and, subsequently, for impaired economic performance as body weight gain and feed conversion ratio [5]. Amprolium, Diclazuril, and Salinomycin are used commonly in Iran. In order to prevent drug resistance, rotation of coccidiostats and shuttle programs are essential [5].

The existence of genetic variation in resistance to coccidiosis among breeds and strains has been reported [19]. In previous studies from Ethiopia and North of Iran, no association was found between infection rate and breed, similar to our work (Table 1) [4, 9, 12]. By attention to less study of breed, planning and conducting extensive research on the impact and role of different breeds in the disease prevalence are essential. Evaluation of parasitic resistance in different breeds of poultry and selection of superior breed may play a significant role in reducing economic losses.

Epithelium damage caused by* Eimeria* is a major predisposing factor for necrotic enteritis (NE), allowing* Clostridium perfringens* to replicate rapidly and produce toxin, probably because leakage of proteins including plasma into the lumen of the gut during* Eimeria* infection provides the protein-rich nutrient substrates favorable to* C. perfringens* proliferation and toxin production. For these reasons,* Eimeria* spp. have often been used in conjunction with* C. perfringens* to induce NE experimentally [20, 21]. In current study, infection rate in farms with history of clostridiosis was 50% (Table 1, OR = 2.6); a significant relationship was observed similar to the finding of previous studies [20, 21]. Coccidiosis can play a significant role in the occurrence of NE when a sufficient number of toxigenic strains of* C. perfringens* type A are present [9, 21].

Colibacillosis occurs as an acute fatal septicemia or subacute pericarditis, airsacculitis, salpingitis, and peritonitis. It is a common disease with global distribution [22]. In this study, there was an increase of 16.5-fold of infection rate in farms with history of colibacilosis (Table 1), which is in line to other hand [22].

Dysentery is a serious sign in clinical coccidiosis [10]. In the present study, 75% of chicks with dysentery were positive (*P* < 0.0001). Our results taken with previous investigations are consistent with the idea that the coccidiosis rate correlated with dysentery.

Different hygiene conditions and management of the anticoccidial programs in farms, study design, methods, and different geographical regions may be the main cause of varied results.

## 4. Conclusions

This is the first report of coccidiosis rate in broiler farms in western Iran. Coccidiosis may be an important factor in the economic losses of the broiler chicks in this region. Therefore, further investigations and design appropriate control strategies in improving management of farms are necessary and strongly recommended.

## Figures and Tables

**Table 1:** Prevalence rate of coccidiosis in broiler farms in different variables in Hamedan province, western Iran.

	Age groups (weeks)			Breed			HA	HC	HCL	HD	Total
	<4	4–6	>6	AAP	CO	RO					
Number of samples (%)	30 (13.6)	80 (36.4)	110 (50)	20 (9.1)	30 (13.6)	170 (77.3)	90 (40.9)	100 (45.5)	40 (18.2)	40 (18.2)	220 (100)
Number of positive (%)	0 (0)	30 (37.5)	40 (36.4)	10 (50)	10 (33.3)	50 (29.4)	60 (66.7)	60 (54.5)	20 (50)	30 (75)	70 (31.8)

AAP: Arbor-Acers-Plus, CO: Cobb, RO: Ross, HA: history of coccidiostats consumption, HC: history of colibacilosis in flock, HCL: history of clostridiosis in flock, and HD: history of dysentery.

**Table 2:** Prevalence rate of different *Eimeria* species in broiler farms in Hamedan province, western Iran.

*Eimeria* spp.	Number of positive	Positive %
*E. acervulina *	53	75.7
*E. tenella *	38	54.3
*E. necatrix *	20	28.6
*E. maxima *	14	20
